# Patterns of inflammation, microstructural alterations, and sodium accumulation define multiple sclerosis subtypes after 15 years from onset

**DOI:** 10.3389/fninf.2023.1060511

**Published:** 2023-03-23

**Authors:** Antonio Ricciardi, Francesco Grussu, Baris Kanber, Ferran Prados, Marios C. Yiannakas, Bhavana S. Solanky, Frank Riemer, Xavier Golay, Wallace Brownlee, Olga Ciccarelli, Daniel C. Alexander, Claudia A. M. Gandini Wheeler-Kingshott

**Affiliations:** ^1^NMR Research Unit, Queen Square MS Centre, Department of Neuroinflammation, UCL Queen Square Institute of Neurology, Faculty of Brain Sciences, University College London, London, United Kingdom; ^2^Radiomics Group, Vall d'Hebron Institute of Oncology, Vall d'Hebron Barcelona Hospital Campus, Barcelona, Spain; ^3^Centre for Medical Image Computing, Department of Medical Physics and Biomedical Engineering, University College London, London, United Kingdom; ^4^eHealth Center, Universitat Oberta de Catalunya, Barcelona, Spain; ^5^Mohn Medical Imaging and Visualization Centre, Department of Radiology, Haukeland University Hospital, Bergen, Norway; ^6^Department of Brain Repair and Rehabilitation, UCL Queen Square Institute of Neurology, Faculty of Brain Sciences, University College London, London, United Kingdom; ^7^NIHR UCLH Biomedical Research Centre, London, United Kingdom; ^8^Department of Brain and Behavioural Sciences, University of Pavia, Pavia, Italy; ^9^Brain Connectivity Research Center, IRCCS Mondino Foundation, Pavia, Italy

**Keywords:** MRI, multiple sclerosis, quantitative, multi-modal, diffusion, sodium, machine learning, random forest

## Abstract

**Introduction:**

Conventional MRI is routinely used for the characterization of pathological changes in multiple sclerosis (MS), but due to its lack of specificity is unable to provide accurate prognoses, explain disease heterogeneity and reconcile the gap between observed clinical symptoms and radiological evidence. Quantitative MRI provides measures of physiological abnormalities, otherwise invisible to conventional MRI, that correlate with MS severity. Analyzing quantitative MRI measures through machine learning techniques has been shown to improve the understanding of the underlying disease by better delineating its alteration patterns.

**Methods:**

In this retrospective study, a cohort of healthy controls (HC) and MS patients with different subtypes, followed up 15 years from clinically isolated syndrome (CIS), was analyzed to produce a multi-modal set of quantitative MRI features encompassing relaxometry, microstructure, sodium ion concentration, and tissue volumetry. Random forest classifiers were used to train a model able to discriminate between HC, CIS, relapsing remitting (RR) and secondary progressive (SP) MS patients based on these features and, for each classification task, to identify the relative contribution of each MRI-derived tissue property to the classification task itself.

**Results and discussion:**

Average classification accuracy scores of 99 and 95% were obtained when discriminating HC and CIS vs. SP, respectively; 82 and 83% for HC and CIS vs. RR; 76% for RR vs. SP, and 79% for HC vs. CIS. Different patterns of alterations were observed for each classification task, offering key insights in the understanding of MS phenotypes pathophysiology: atrophy and relaxometry emerged particularly in the classification of HC and CIS vs. MS, relaxometry within lesions in RR vs. SP, sodium ion concentration in HC vs. CIS, and microstructural alterations were involved across all tasks.

## 1. Introduction

Multiple sclerosis (MS) is an immune-mediated, inflammatory, neurodegenerative disease of the central nervous system characterized by inflammatory demyelination and heterogeneous accrual of physical disability (Lucchinetti et al., 2000). The onset is determined by the first inflammatory episode suggestive of MS, referred to as clinically isolated syndrome (CIS), with CIS being recognized as the first clinical instance in the MS spectrum (Lublin et al., 2014). Further neurological symptoms may lead to a clinically defined diagnosis, as determined by the updated McDonald criteria (Thompson et al., 2018). Based on the clinical course, patients can be categorized into three types of relapse-onset MS: CIS, relapsing remitting (RR) and secondary progressive (SP). RR is characterized by clinically defined focal activity followed by periods of total or partial remission of neurological deficit, and the lack of disease progression between attacks; SP may follow from an initial RR course, with progressive worsening of neurological symptoms, with or without acute relapses. Primary progressive MS is associated with a progressive deterioration of clinical symptoms from onset (Lublin et al., 2014). Understanding why patients may develop different MS phenotypes over the years, or why only a small fraction of the diversity of clinical disability in MS can be explained by radiological evidence (*clinico-radiological paradox*) (Barkhof, 1999, 2002), are cause for further research.

Magnetic resonance imaging (MRI) is instrumental in the diagnosis and prognosis of MS, routinely used in clinical practice for the acquisition of qualitative images, e.g., proton density- (PD), T2- and T1-weighted, for lesion assessment. In the research environment, a much wider spectrum of dedicated, quantitative MRI techniques are employed for the study and characterization of MS pathophysiology, investigating the complex relationship between radiological evidence and clinical disability (Chard and Trip, 2017; Filippi et al., 2019). *In vivo* imaging biomarkers can be sensitive to inflammation, microstructural alterations, and even sodium ions accumulation, providing a window into the disease pathophysiology over time. Brain atrophy is a known indicator of disease progression since the early stages of MS, with recent studies providing further insight into the hierarchical recruitment of different brain regions over time (Eshaghi et al., 2018), although the integration of specifically cortical and sub-cortical regional volumetric measurements in clinical practice has yet to reach a consensus (Sastre-Garriga et al., 2020). Relaxometry and quantitative PD imaging have been shown to provide good biomarkers for inflammation and demyelination in normal appearing tissue (Neema et al., 2007; Mezer et al., 2013), invisible to the standard qualitative imaging. Through sensitivity to the diffusion of water molecules within the structured axonal environment of the brain, diffusion weighted imaging (DWI) has shown microstructural alterations in both lesions and normal appearing tissues, correlating with physical disability in progressive MS (Filippi et al., 2001; Collorone et al., 2020); recent studies have reported abnormalities at the early stages (Tur et al., 2020) and potential links to cognitive disability as well (Savini et al., 2019). Sodium (^23^Na) imaging has been used to access the signal induced by sodium ions, showing promise in probing axonal function directly (Gandini Wheeler-Kingshott et al., 2018), with evidence of increased total sodium concentration (TSC) being reported in MS, correlating with disability and disease progression (Inglese et al., 2010; Paling et al., 2013; Maarouf et al., 2014). Whilst the potential of advanced MRI modalities is evident, they lack a unified consensus about their implementation, optimization and interpretation, and require, when compared to standard routine scans, additional acquisition times, costs and expertise, which make their application in clinics limited.

In this work, we explored a multi-modal dataset acquired in a cohort of patients with the same disease duration, where clinical and MRI assessments were performed 15 years from CIS, comprehending both routine and advanced MRI metrics sensitive to inflammation, microstructural alteration and sodium ions accumulation. Using a machine learning approach, we aimed to gain further understanding of which modalities are more likely to carry biophysically meaningful information for different classification tasks. Machine learning indeed has shown to be a key tool in the data-driven exploration of MRI datasets for the identification of patterns and biomarkers of disease, including the ability to identify discriminating factors of disease phenotypes against each other and healthy controls (HC) (Wottschel et al., 2015; Eshaghi et al., 2016). We therefore trained and tested a *random forest* algorithm to classify different subtypes of MS vs. HC and between each other, using a rich array of quantitative imaging features extracted from both clinical and advanced MRI data. Feature importance was calculated for each task and used to assess which metrics mostly contributed to the decision-making process. This provided us with novel insights into the pathophysiology of different MS subtypes, while also informing future studies toward more task-efficient MRI acquisitions.

## 2. Methods

A retrospective (Brownlee et al., 2019) multi-modal MRI dataset of HC, CIS, RR, and SP patients with same disease duration was analyzed to provide evidence on what MRI features are best representative for different classification tasks.

### 2.1. Cohort

The cohort consisted of a total of 123 subjects: 29 HC (10 men, age: 35 ± 10 years old), 18 CIS (6 men, age: 47 ± 10 years old, EDSS: 0.4 ± 0.5), 63 RR (15 men, age: 47 ± 8 years old, EDSS: 2.2 ± 1.1), and 13 SP (4 men, age: 48 ± 8 years old, EDSS: 5.5 ± 1.2). All MS patients (CIS, RR, SP) attended the MS center for clinical and radiological follow-up after a mean of 15 years from onset (Brownlee et al., 2019).

### 2.2. MRI protocol

Data were acquired on a 3T Philips Achieva MR system. The acquisition protocol included:

1. **PD/T2-w**. Dual-echo 2D PD/T2-weighted turbo spin-echo (resolution: 1 × 1 × 3mm^3^, echo time TE: 19/85m, repetition time TR: 3,500ms, turbo factor: 10, echo spacing: 9.4ms, scan time: 4′2″).
2. **T1-w**. 2D T1-weighted spin-echo (resolution: 1 × 1 × 3mm^3^, TE: 10ms, TR: 625ms, scan time: 5′43″).
3. **DWI**. Cardiac-gated, multi-shell, diffusion-weighted echo-planar imaging, with {8, 15, 30} isotropically distributed directions at *b*-values: {300, 711, 2000} s/mm^2^ (resolution: 2.3 × 2.3 × 2.3mm^3^, TE: 82ms, nominal TR (12 heart-beats): 13846ms, scan time: ~16′).
4. **Sodium**. ^23^Na imaging with 3D-cone sampling trajectory (resolution: 3 × 3 × 3mm^3^, TE: 0.22ms, TR: 120ms, scan time: ~18′). Two 4% agar phantoms with sodium concentration of 40 and 80mM were placed near the subject's head during the image acquisition for calibration purposes (Riemer et al., 2014).
5. **3DT1**. 3D sagittal T1-weighted magnetization-prepared rapid gradient echo (resolution: 1 × 1 × 1mm^3^, TE: 3.1ms, TR: 6.9ms, inversion delay time: 823ms, flip angle: 8′, scan time: 6′32″).

All proton scans were acquired using a 32 channel head coil, whilst sodium imaging was performed using a single channel transmit-receive volume head coil (Rapid Biomedical, Rimpar, Germany). Patients were repositioned prior to the sodium imaging scans to allow for the coil change.

### 2.3. Image analysis

Lesion masks from Brownlee et al. (2019) studies were used. Brain tissue segmentation was performed on lesion-filled (Prados et al., 2016a) 3DT1 using the *Geodesic Information Flows* (GIF) tool (Cardoso et al., 2015), obtaining masks of white matter (WM), deep gray matter (dGM), and cortical gray matter (cGM).

PD/T2-weighted scans were initially acquired for lesion segmentation only; however, given the availability of T1-weighted scans with similar readout, they were also used to extract quantitative estimates of PD, T2, and T1 maps by fitting the relevant Bloch equations, using the *MyRelax* toolbox (Grussu et al., 2020). Further details are reported in the Supplementary material.

DWI data were corrected for motion and eddy current distortion using *FSL* (Andersson and Sotiropoulos, 2016). The *spherical mean technique* (SMT) multi-compartment model (Kaden et al., 2016) was used to analyse the DWI data, producing maps of intra-neurite volume fraction, intrinsic diffusivity and orientation dispersion entropy.

TSC maps were calculated by calibrating the ^23^Na images by the signal intensity within the phantoms (Inglese et al., 2010; Riemer et al., 2014), which were segmented automatically (Prados et al., 2016b).

Mean values for quantitative PD, T2, and T1, intra-neurite volume fraction, intrinsic diffusivity, entropy, and TSC were calculated in normal appearing white matter (NAWM), dGM, cGM, and lesions, when present. Details about the calculation of the summary statistics are reported in the Supplementary material. The volume for the three tissue classes (WM, dGM, cGM) was also calculated from the brain segmentation, and divided by the total intra-cranial volume to take into account variability in head-sizes. In total, for each of the 123 subjects, 31 regional variables, or *biophysically meaningful features*, were therefore calculated.

Due to the HC group being significantly younger than the rest (average age 12 years lower, *p* = 1 × 10^−6^ from Kruskal–Wallis test), all features other than lesions-based ones were corrected for age using the HC as reference. A linear model, with age as independent variable and {β_0_, β_1_} as intercept and slope, respectively, was fitted feature-wise on the HC data: the features that resulted significantly (*p* < 0.05) correlated with age were corrected by subtracting β_1_ × age from the original data.

### 2.4. Classification analysis

After correcting for age, the data was *standardized* feature-wise such that the value distribution for each feature had mean of zero and standard deviation of one. The dataset was used to train and test a random forest algorithm over different binary classification tasks: HC vs. MS (that is RR and SP), CIS vs. MS, and all binary permutations of HC, CIS, RR, and SP. All HC and most CIS had no lesions, therefore lesion features were included only for the RR vs. SP classification task.

Classification was implemented using Python 3.7.4 (VanRossum and Drake, 2010) and the *scikit-learn* package (Pedregosa et al., 2011). Default parameters for the ensemble.RandomForestClassifier function were selected, with the number of trees set to 1,000 based on the available literature and previous experience on datasets with similar dimensionality. For each classification task, a 10-fold stratified cross-validation with 10 repetitions was implemented, for a total of 100 iterations. The classification performance was assessed by the average *receiver operating characteristic* (ROC) *area under the curve* (AUC) score on the test set across the 100 train/test iterations. Variable importance is defined by the improvement in the split-criterion attributed to each variable (feature) during training of the random forest. Variable importances were averaged across iterations, returning the mean feature ranking for the task; this allowed to identify the features that most contributed to each classification task, and thus are more likely to be biophysically meaningful with respect to the groups characterization.

In order to assess the significance of the classification results, the training and testing process was repeated identically 1,000 times with randomly permuted labels of the subjects at each repetition. The distribution of the 1,000 mean ROC AUC scores defined the random classifier performance profile, which was used as reference to calculate the *p*-value associated to the classification performances on the original data.

## 3. Results

### 3.1. Age correction

**Figure 1:**
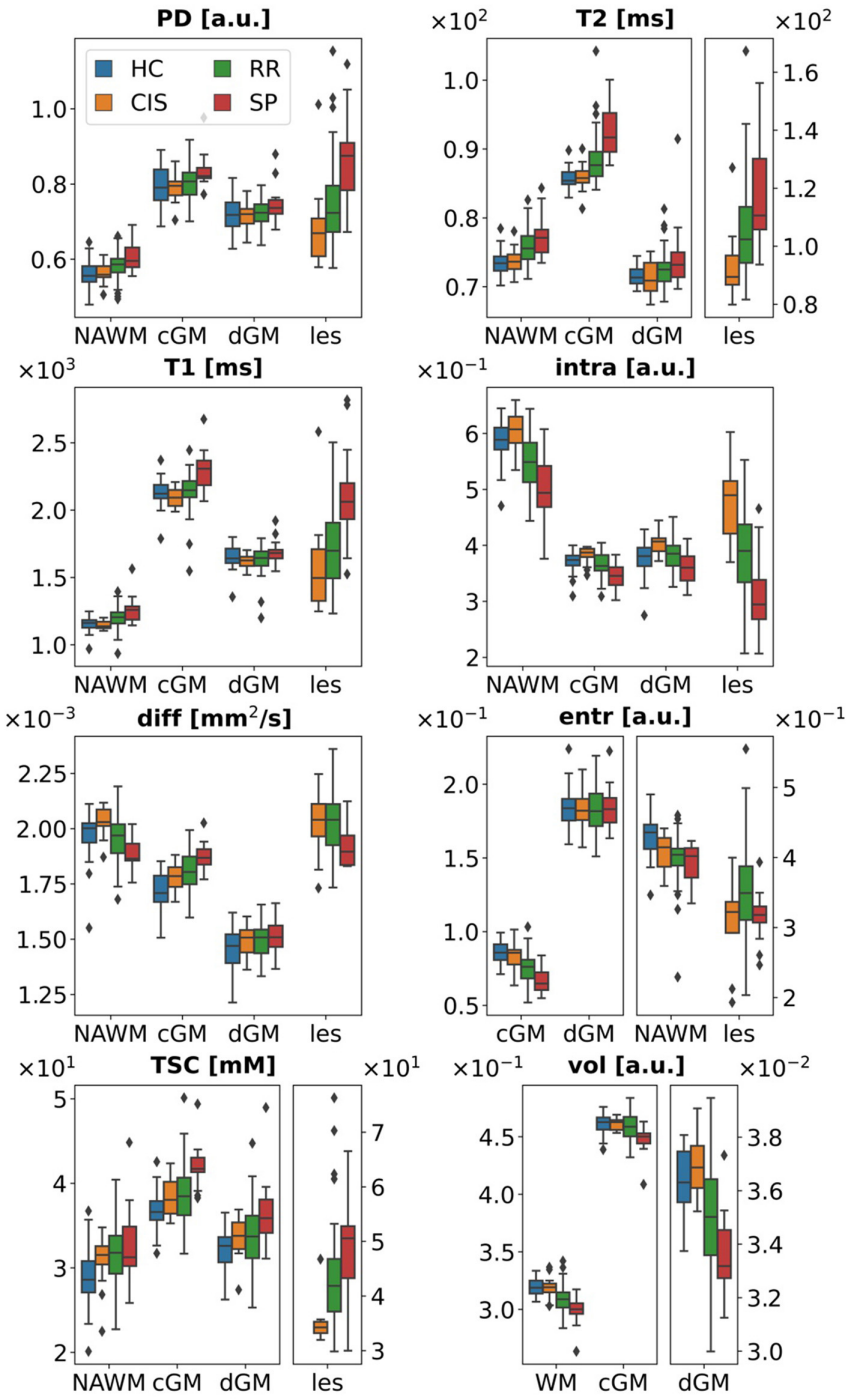
Age-corrected data. Data points for some tissue types have been plotted against different *y*-axes to better visualize boxplots across different ranges. PD, proton density; intra, intra-neurite volume fraction; diff, intrinsic diffusivity; entr, orientation dispersion entropy; TSC, total sodium concentration; vol, tissue volume; WM, white matter; NAWM, normal appearing white matter; cGM, cortical gray matter; dGM, deep gray matter; les, lesions; a.u., arbitrary units.

Of the 24 non-lesion features, 5 resulted significantly correlated with age: quantitative T2 in dGM (β_1_ = −0.09, *p* = 0.002), and cGM (β_1_ = −0.07, *p* = 0.02), intrinsic diffusivity in dGM ($\beta_{1}=5\times 10^{-6}$, *p* = 0.02), volume of dGM ($\beta_{1}=6.6\times 10^{-5}$, *p* = 0.006) and cGM ($\beta_{1}=4.8\times 10^{-4}$, *p* = 0.009). Fitting results for all features are reported in the Supplementary material. Age-corrected feature distributions are shown in Figure 1.

### 3.2. Classification results

ROC AUC scores for each task are reported in Table 1. In addition to the mean, the median and interquartile range [*Q*_1_, *Q*_3_], with *Q*_1,3_ indicating the 25-th and 75-th percentiles respectively, are also reported to assess dispersion instead of standard deviation, as the ROC AUC distribution over the 100 iterations was not symmetric, but skewed toward better-than-chance performance values. The best classification performances were obtained for the HC vs. SP and CIS vs. SP tasks, with mean ROC AUC scores of 0.99 and 0.95, respectively. Mean ROC AUC scores for HC vs. RR and CIS vs. RR were 0.82 and 0.83, and when discriminating HC and CIS against both the clinically defined MS groups, the performance scores fell in between. The lowest scores were observed for the RR vs. SP and HC vs. CIS tasks, with mean ROC AUC scores of 0.76 and 0.79, respectively. Mean ROC AUC, sensitivity and specificity scores have been also calculated with an random under-sampling method to correct for group imbalance, and reported in the Supplementary material.

**Table 1:** ROC AUC classification results.

**Tasks**	**Mean**	**Median [*Q*_1_, *Q*_3_]**	***p*-value**
HC - RR	0.82	0.83[0.72, 0.94]	< 0.001
HC - SP	0.99	1.00[1.00, 1.00]	< 0.001
HC - MS	0.84	0.88[0.76, 0.92]	< 0.001
CIS - RR	0.83	0.89[0.75, 1.00]	< 0.001
CIS - SP	0.95	1.00[1.00, 1.00]	< 0.001
CIS - MS	0.84	0.87[0.71, 1.00]	< 0.001
RR - SP	0.76	0.83[0.67, 1.00]	< 0.01
HC - CIS	0.79	0.83[0.67, 1.00]	< 0.01

*p*-values calculated through permutation test. HC, healthy controls; CIS, clinically isolated syndrome; RR, relapsing-remitting MS; SP, secondary progressive MS; MS, RR and SP; *Q*_1, 3_, 1st, 3rd quartile, respectively.

### 3.3. Permutation test

**Figure 2:**
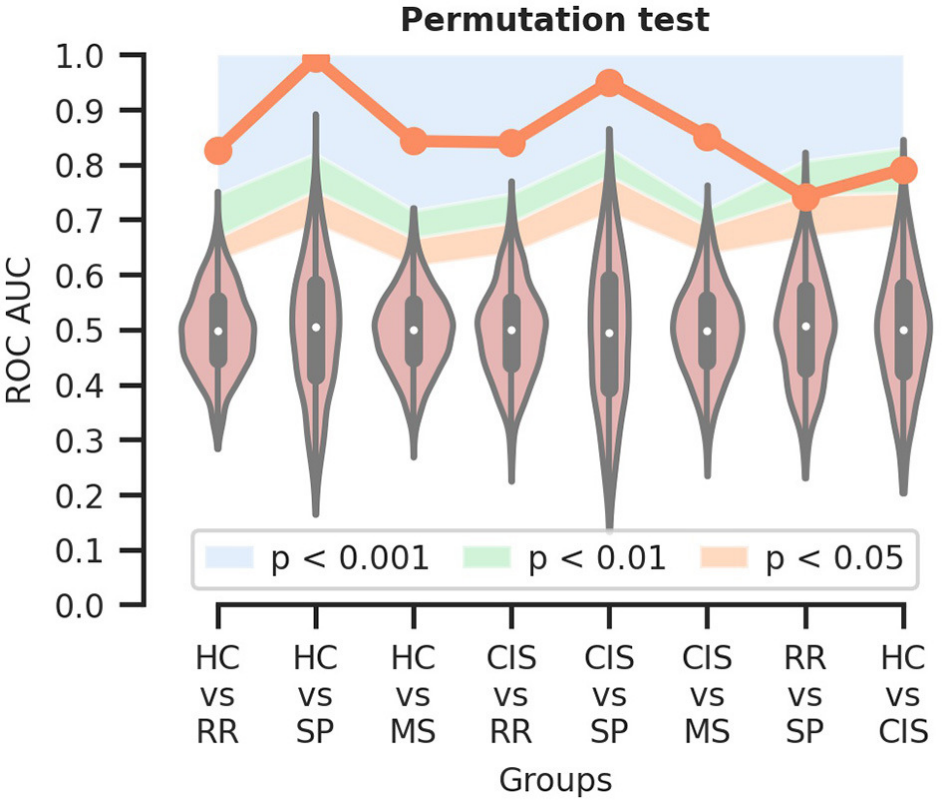
Permutation test to assess the statistical significance of the classification results. The orange line indicates the mean ROC AUC for each classification task; the colored areas delineate the different ranges of significance. HC, healthy controls; CIS, clinically isolated syndrome; RR, relapsing-remitting MS; SP, secondary progressive MS; MS, RR and SP.

The random classifier performance profiles for the different tasks are shown in Figure 2. Statistical significance of 0.001 < *p* < 0.01 was observed for the RR vs. SP and HC vs. CIS classification tasks, whilst *p* < 0.001 was recorded for all others.

### 3.4. Feature importance

**Figure 3:**
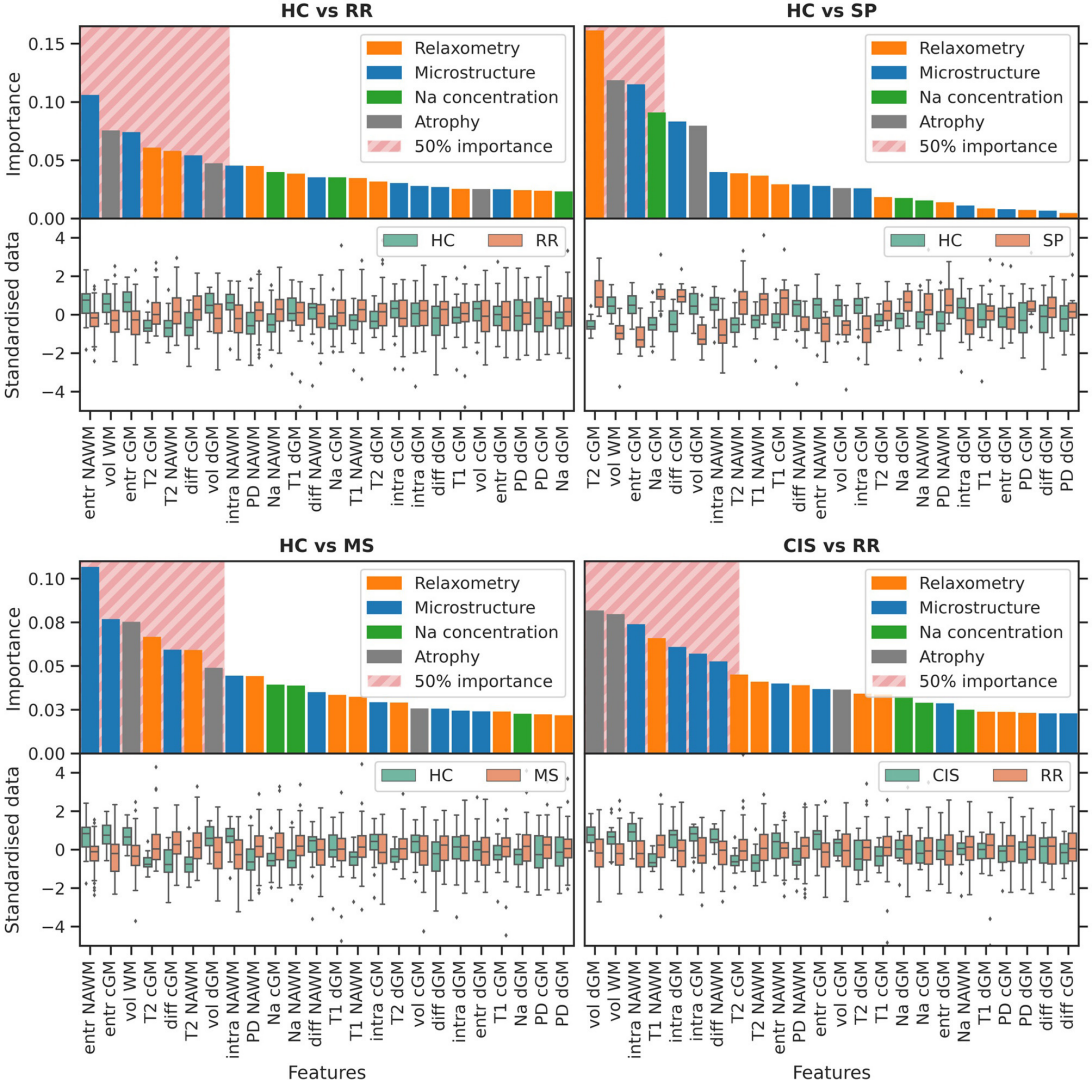
Variable importance. The bar-plot is color-coded to group MRI modalities from the same biophysical source. The striped block highlights the features contributing to 50% of the classification process. The *y*-axis is shared between columns. PD, proton density; intra, intra-neurite volume fraction; diff, intrinsic diffusivity; entr, orientation dispersion entropy; TSC, total sodium concentration; vol, tissue volume; WM, white matter; NAWM, normal appearing white matter; cGM, cortical gray matter; dGM, deep gray matter; les, lesions; HC, healthy controls; CIS, clinically isolated syndrome; RR, relapsing-remitting MS; SP, secondary progressive MS; MS, RR and SP. Continuing to Figure 4.

**Figure 4:**
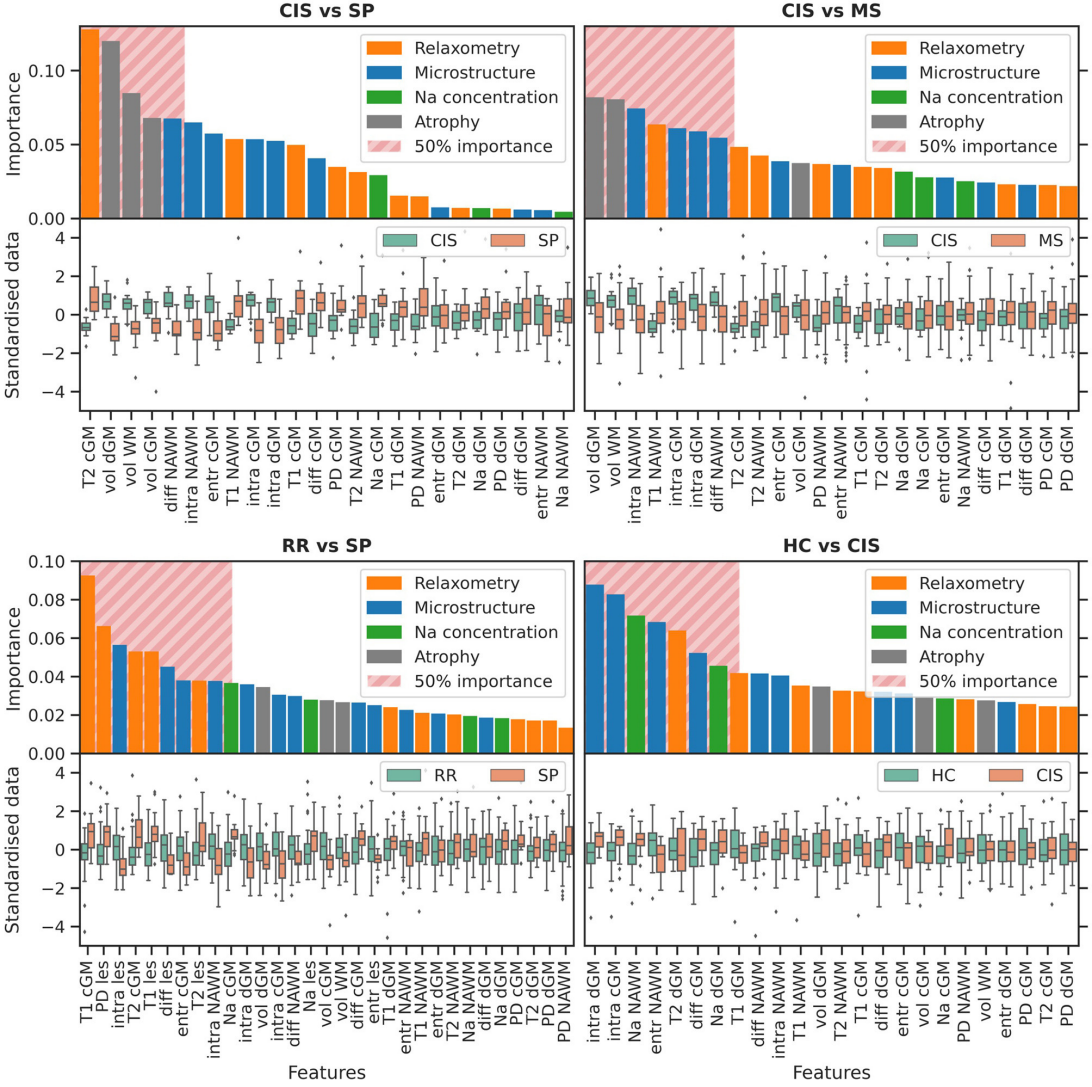
Variable importance. Continued from Figure 3.

Average feature ranking for all classification tasks is shown in Figures 3, 4. Features have been color-coded to group MRI modalities from the same biophysical source (e.g., relaxometry parameters are in orange, diffusion microstructure in blue, sodium concentrations in green and tissue volume in gray); the top-ranking features contributing to 50% of the decision process for each task have been highlighted by a striped block and considered for interpretation. Overall, tissue volumes were the most meaningful when discriminating HC and CIS against clinically-defined MS; relaxometry parameters mainly in lesions had a role when discriminating the clinically-defined MS subtypes against each others; diffusion metrics were meaningful across all tasks, although particularly prominent in differentiating CIS vs. HC; TSC was mostly relevant when discriminating CIS against HC.

- **HC vs. RR**. RR patients showed a reduced WM and dGM volume, as well as reduced orientation dispersion entropy and increased T2 in NAWM and cGM with respect to HC. Increased intrinsic diffusivity in cGM also contributed to the classification task, with reduced intra-neurite volume fraction in NAWM at the 50% cumulative importance threshold.
- **HC vs. SP**. The decision task was mostly driven by increased T2 in cGM, and reduced WM volume and entropy in cGM of SP compared to HC. Increased TSC and diffusivity in cGM were also observed in SP at the 50% threshold.
- **HC vs. MS**. Top-ranking features were distributed similarly to the HC vs. RR task.
- **CIS vs. RR**. Reduced dGM and WM volume mostly characterized RR compared to CIS, together with reduced intra-neurite volume fraction across all tissues, and increased T1 in NAWM. Reduced intrinsic diffusivity also emerged in NAWM in RR.
- **CIS vs. SP**. The task was driven mostly by reduced volume of all tissues in SP, and increased T2 in cGM. Reduced diffusivity and intra-neurite volume fraction in NAWM was also observed at the 50% threshold in SP compared to CIS.
- **CIS vs. MS**. Similar top-ranking features to the CIS vs. RR task were observed.
- **RR vs. SP**. The task was driven mostly by relaxometry—increased PD, T2 and T1—and diffusion metrics—reduced intra-neurite volume fraction, diffusivity—alterations in lesions in SP compared to RR. Increased T1, T2 and entropy in cGM were also observed, with increased TSC in NAWM at the 50% threshold.
- **HC vs. CIS**. Increased TSC in NAWM and dGM in CIS compared to HC appeared as top-ranking features, together with increased intra-neurite volume fraction in dGM and cGM, reduced entropy in NAWM, and increased diffusivity in cGM. Reduced T2 and T1 in dGM were also observed in CIS.

## 4. Discussion

In this work, we used random forest classifiers to study the interaction between features extracted from both routine scans and advanced diffusion and sodium weighted imaging for the purpose of characterizing CIS and clinically-defined MS subtypes. The results show that a combination of advanced quantitative MRI and clinical features achieve classification scores between 76% and 99% depending on the task. Moreover, it is apparent that after 15 years from the initial CIS episode, features reflecting inflammation, microstructure changes and sodium accumulation play a very different role in each MS subtype. Whether these alterations are present from the start or are the result of a 15 years evolution, it is not possible to say from this dataset, and requires targeted longitudinal studies.

- **Atrophy**. Brain atrophy was observed in the MS groups and emerged as a meaningful feature in all the classification tasks discriminating MS patients against HC and CIS. No strong involvement of brain volume features was observed instead when classifying MS phenotypes against each other, and in the HC vs. CIS task. Tissue volume loss in MS showed heterogeneous behavior across the different tissues, particularly with dGM consistently scoring higher than cGM, which is in line with previous findings of progressive recruitment of gray matter structures as part of MS neurodegeneration (Eshaghi et al., 2018; Soares et al., 2020). dGM significant involvement is well-known in the scientific community, however a consensus for the incorporation of *global* gray matter volumetrics into clinical practice has only recently been reached, and the specific inclusion of dGM structures (e.g., thalami, basal ganglia) in particular is still debated (Sastre-Garriga et al., 2020). Further research is recommended, with this work providing evidence in this direction.
- **Relaxometry**. Relaxometry features contributed partially to most tasks involving the clinically defined MS population, with prolonged T2 in cGM emerging particularly when discriminating SP against HC and CIS, a possible biomarker for the advanced cortical demyelination observed in the progressive stages of MS (Magliozzi et al., 2018). The strongest contribution was however observed in the RR vs. SP classification task across all relaxometry features, both in cGM and lesions: the involvement of quantitative parameters measured in lesions in the discrimination of MS phenotypes is indicative of the heterogeneous nature of MS pathogenesis and neurodegeneration (Lucchinetti et al., 2000). This result supports the need for adopting a more quantitative approach to lesion characterization in clinics than mere lesion load assessment. Reduced T2 in dGM was also observed in CIS with respect to HC: this reduction goes against a possible demyelination effect and could be due to residual (after age correction) iron deposition (Aquino et al., 2009). Further studies of iron deposition in MS, using for example quantitative susceptibility mapping and magnetic susceptibility source separation approaches, are recommended (Shin et al., 2021).
- **Diffusion imaging**. Diffusion imaging metrics were involved in all classification tasks, which is expected given that microstructural alterations are at the core of MS demyelination and neurodegeneration. Despite being the most ubiquitous set of feature across all tasks, diffusion metrics from multi-compartment models are also strongly model-dependent, hence prone to modeling artifacts and limitations, e.g., the lack of a myelin compartment, and results should be interpreted with care. Against HC, MS patients exhibited overall reduced orientation dispersion entropy, and reduced intra-neurite volume fraction against CIS. These results are in line with findings of reduced *fractional anisotropy* associated to higher fiber dispersion and neurite loss (Roosendaal et al., 2009). Reduced intrinsic diffusivity in SP was also observed with respect to CIS and RR, both in NAWM and lesions, but not in gray matter, which may be spurious, or an indicator of new lesion formation compatible with axonal undulation (Grussu et al., 2016). Increased intra-neurite volume fraction in CIS emerged in the classification against HC: whilst counter-intuitive in the context of neurite loss, this may be suggestive of axonal swelling, as further discussed below.
- **Sodium imaging**. TSC was particularly meaningful when discriminating CIS vs. HC, with increased TSC being observed in CIS in NAWM and dGM. Increased TSC was also observed in MS patients, albeit with a lower contribution with respect to other features, and has been reported in literature from the early stages of the disease (Maarouf et al., 2017). It has been associated with the over-expression and redistribution of sodium-potassium channels from the Ranvier nodes to newly demyelinated membrane: this is an adaptive response to the disruption of saltuatory conduction caused by demyelination, apt to preserve action potential transmission, limit the onset of neurological deficits, and facilitate recovery. This however also increases the axonal metabolism, as the proliferation of the sodium-potassium *active* pumps comes with higher energy expenditure which, if not satisfied, causes the accumulation of intra-cellular sodium. In MS, the impaired trophic support from oligodendrocites and mitochondrial dysfunction contribute to energy under-production which, coupled with the increased metabolic need, can lead to axonal degeneration due to metabolic failure secondary to chronic energy deprivation (Petracca et al., 2016). In the case of CIS, the increased TSC might be explained as a long lasting effect established in the brain following the initial inflammatory event. It may be speculated that this might be due to an over-expression of sodium channels at the onset of CIS to support neuronal function, which may also explain the increased intra-neurite volume fraction detected with diffusion imaging: indeed, the intra-cellular accumulation of sodium might induce axonal swelling through osmosis (Armstrong, 2003). To what extent this can happen before functional derangement accrues, leading to a more severe MS phenotype, is to be investigated.

Overall, the results of this unique dataset with MS patients of same disease duration, and a rich multi-modal quantitative MRI protocol, have shown that atrophy and relaxometry features contribute significantly to the discrimination of MS patients from HC and CIS; relaxometry in lesions emerges as particularly involved in the classification of MS phenotypes, which highlights the heterogeneity of MS pathophysiology. With both brain volumetry and relaxometry features being extracted from routine scans readily available in clinical practice, we have offered evidence of the hidden potential qualitative MRI data holds beyond lesion and tissue segmentation. Whilst advanced MRI acquisitions ought to be preferred when available, they are far from being routinely introduced in clinics; on the other hand, the use of routine scans can pave the way to quantitative studies on large historical datasets otherwise lacking dedicated quantitative modalities. Advanced diffusion and sodium imaging have proven particularly sensitive to the characterization of MS phenotypes against each other, and CIS against HC, where differences in atrophy or relaxometry scores in normal appearing tissues were not as important, or not present at all. In these cases, dedicated quantitative MRI modalities showed their role in the quantification of subtle tissue microstructural and physiological alterations, otherwise invisible to conventional MRI, offering further insights on MS heterogeneous neurodegeneration. Specifically, CIS presenting subtle alterations compatible with MS histopathology (sodium ions accumulation and possible axonal swelling) may mark long lasting subtle damage accrued as a result of the first episode of neurologic symptoms. Alternatively, the observed alterations suggest that neuroprotective mechanisms may be at play in the stable CIS population, but, unlike with the clinically-defined MS patients, they do not lead to meaningful atrophy, inflammation, demyelination, and axonal loss. In other words, the ability to adapt to the increased metabolic demand without succumbing to energy failure, or avoiding axonal degeneration by excessive osmotic swelling, might be compensatory or even protective mechanisms, and as such key factors in what determines conversion, or lack thereof, to clinically defined MS.

Interestingly, what differentiates RR from a progressive form of the disease characterizing SP are changes in relaxometry parameters in the lesions. The classification task ranked as highest not microstructure changes or sodium accumulation in normal appearing tissue, but alterations of relaxometry parameters in the lesions of SP patients compared to RR. This could give an insight into the possible source of disease progression, driven not by the number or volume of the lesions, nor by diffuse damage of tissue, but by the severity and biophysical nature of lesion alterations. This therefore calls on monitoring relaxometry, as well as potentially others quantitative biophysically meaningful features, in the lesions as potential predictor of risks of progression.

The interpretation of these findings is of course conditional on this study's limitations. The statistical significance is hindered by the small sample size, especially tasks involving the SP group—only 13 subjects: although spurious results due to the many features may be expected, we strove to minimize their impact on the final outcome through careful examination of the data and corroboration with the published literature. In terms of classification tasks, the class imbalance between RR and SP likely caused the RR group to drive the classification results when discriminating HC or CIS against the whole MS cohort (RR and SP). Feature selection was performed implicitly by the random forest based on the relative contribution of each feature to the classification. No prior feature selection was performed as it would have reduced the exploratory power of this study. Each MRI modality came with its own limitations, which also must be taken into consideration, e.g., the multi-compartment diffusion model lacking a myelin volume fraction, or the use of surrogate quantitative PD, T2, and T1 extracted from routine scans not optimized to the scope. Particularly, the *MyRelax* algorithm for the calculation of quantitative T1 diverges in regions of cerebrospinal fluid, which were not of interest to this work; however, lesions also exhibit a similar behavior at their core, therefore the summary statistics for quantitative T1 in lesions is to be intended as representative more of the peripheral part of lesions, where the partial volume effect with cerebrospinal fluid is less pronounced, than the central part. Additional studies with larger sample size and histological evidence are required to substantiate these findings.

We showed that different MRI features appear to be biophysically meaningful when discriminating CIS and clinically defined MS phenotypes, with qualitative and quantitative MRI modalities offering specific insights for different classification tasks. Key to our results is highlighting the need for further studies focused on the role of quantitative MRI in the lesions of early CIS and MS subjects to score risks of progression. These findings can help in further understanding MS pathophysiology, as well as inform future studies toward more efficient acquisition protocols, better tailored to the scope.

## Data availability statement

The raw data supporting the conclusions of this article will be made available by the authors, without undue reservation.

## Ethics statement

The studies involving human participants were reviewed and approved by Research Ethics Committee 13/LO/1413. The patients/participants provided their written informed consent to participate in this study.

## Author contributions

AR, OC, DA, and CGWK designed the study. WB enrolled the subjects and collected all the clinical data. MY acquired the MRI data, with support from BS, FR, XG, and CGWK. AR designed and performed the analyses with support from FG, BK, FP, DA, and CGWK. OC and CGWK provided support and guidance with the interpretation of the results. AR wrote the manuscript, with support from OC and CGWK, and comments from all authors. All authors contributed to the article and approved the submitted version.
