# Supplementary material for: Patterns of inflammation, microstructural alterations, and sodium accumulation define multiple sclerosis subtypes after 15 years from onset
Source: Front Neuroinform. 2023 Mar 23;17:1060511. doi: 10.3389/fninf.2023.1060511 (PMC10076673; doi:10.3389/fninf.2023.1060511)
Supplement: Supplementary file 1 [file Data_Sheet_1.pdf]

Supplementary Material

MyRelax. The `getPDT2T1TurboSpinEcho` function of the *MyRelax* toolbox enables to calculate quantitative PD, T2 and T1 maps from PD/T2- and T1-weighted routine scans by solving the associated Bloch equations in each voxel:

$$\begin{aligned} S_{\mathrm{PD}} &= S_0 e^{-\mathrm{TE}_1/T2}(1 - e^{-\mathrm{TR}'_1/T1}) \\ S_{T2} &= S_0 e^{-\mathrm{TE}_2/T2}(1 - e^{-\mathrm{TR}'_1/T1}) \\ S_{T1} &= S_0 e^{-\mathrm{TE}_3/T2}(1 - e^{-\mathrm{TR}_2/T1}) \end{aligned} \tag{S1}$$

where S_{PD}, S_{T2} and S_{T1} are the PD-, T2- and T1-weighted MRI signal in any given voxel. TE and TR are echo and repetition times respectively, and the *effective* $\mathrm{TR}' = \mathrm{TR} - \mathrm{TF} \times \mathrm{ES}$ is used for the PD/T2-weighted scans to correct for the turbo spin-echo acquisition (TF: turbo factor, ES: echo spacing) Rydberg et al. (1995). $S_0 \propto \mathrm{RP} \times \mathrm{PD}$ indicates the *apparent* PD, proportional to the product between quantitative PD and the *bias field* produced by the receiver coil sensitivity profile (RP); bias field correction is performed by making use of the T1 map calculated in the step above, and the known empirical linear correlation between the inverse of T1 and PD Volz et al. (2012):

$$\frac{1}{\mathrm{PD}} \approx A + \frac{B}{T1} \tag{S2}$$

Outliers exclusion. Due to the small size of the dataset, classification results are particularly sensitive to outliers. In order to reduce the chance of spurious results, features distributions were carefully examined prior to calculating summary statistics. To exclude noisy voxels and modelling artifacts, each feature distribution was constrained within biophysically sensible ranges: (0.02, 2) arbitrary units (a.u.) for quantitative PD; (0.5, 500) ms for quantitative T2; (5,5000) ms for quantitative T1; (0.01, 1) a.u. for intra-neurite volume fraction; (0.0008, 0.003) mm^2/s for intrinsic diffusivity; (0.01, 2) a.u. for orientation dispersion entropy; (0.2, 200) mM for TSC. To then reduce the effects of partial volume with cerebrospinal fluid or adjacent tissues, the *interquartile range* $\mathrm{IQR} = Q_3 - Q_1$, with $Q_{1,3}$ being the 25-th and 75-th percentile respectively, was calculated for each features, and voxels with values outside the range ($Q_1 - 1.5 \times \mathrm{IQR}$, $Q_3 + 1.5 \times \mathrm{IQR}$) were excluded.

Age correction. Parameters for the age correction linear model (age independent variable, $\{\beta_0, \beta_1\}$ as intercept and slope) are reported in Table S1. Age correction was performed on the features significantly correlating with age ($p < 0.05$) by subtracting $\beta_1 \times$ age from the original data.

Sensitivity and specificity. Sensitivity and specificity tend to be misleading in imbalanced dataset, as they are driven by the minority/majority group. On the other hand, ROC AUC scores incorporate the information of both sensitivity and specificity in a single score, making the interpretation of the model performance more straightforward, even in imbalanced datasets. To properly calculate sensitivity and specificity, we re-ran the classification tasks by also implementing a random under-sampling step to correct for data imbalance, so that at each

iteration both training and test sets were balanced between groups. Mean ROC AUC, sensitivity and specificity scores for all tasks are reported in Table S2. AUC scores are comparable to those already reported with no under-sampling.

Table S1. Age correction linear model parameters $\{\beta_0, \beta_1\}$. *PD*: proton density; *intra*: intra-neurite volume fraction; *diff*: intrinsic diffusivity; *entr*: orientation dispersion entropy; *TSC*: total sodium concentration; *vol*: tissue volume; *WM*: white matter, *NAWM*: normal appearing white matter; *cGM*: cortical grey matter; *dGM*: deep grey matter.

Feature		β_1	β_0	p-value
PD	NAWM	-0.000125	0.566	0.8605
	dGM	-0.000380	0.734	0.6556
	cGM	-0.000617	0.814	0.5151
T2	NAWM	-0.002141	73.807	0.9508
	dGM	-0.088678	71.495	0.0021
	cGM	-0.069430	85.800	0.0172
T1	NAWM	-0.519758	1170.970	0.6394
	dGM	-1.860150	1716.044	0.2501
	cGM	-3.066843	2235.797	0.1274
intra	NAWM	0.000946	0.550	0.1732
	dGM	0.000964	0.340	0.1047
	cGM	0.000249	0.362	0.5018
diff	NAWM	0.000004	0.002	0.0614
	dGM	0.000005	0.001	0.0158
	cGM	0.000003	0.002	0.0515
entr	NAWM	-0.000768	0.457	0.1640
	dGM	0.000343	0.173	0.2144
	cGM	-0.000123	0.090	0.3551
TSC	NAWM	-0.014404	29.463	0.8365
	dGM	-0.018377	32.711	0.7196
	cGM	0.044006	35.299	0.3421
vol	WM	0.000120	0.315	0.3839
	dGM	-0.000066	0.036	0.0058
	cGM	-0.000484	0.462	0.0093

Table S2. Mean ROC AUC, sensitivity and specificity scores for all classification tasks computed using random under-sampling to correct for group imbalance.

Tasks	ROC AUC	Sensitivity	Specificity
HC - RR	0.83	0.74	0.74
HC - SP	0.99	0.93	0.92
HC - MS	0.84	0.74	0.80
CIS - RR	0.84	0.68	0.81
CIS - SP	0.95	0.76	0.88
CIS - MS	0.85	0.71	0.78
RR - SP	0.74	0.63	0.64
HC - CIS	0.79	0.78	0.64

REFERENCES

Rydberg, J. N., Riederer, S. J., Rydberg, C. H., and Jack, C. R. (1995). Contrast optimization of fluid-attenuated inversion recovery (flair) imaging. *Magnetic resonance in medicine* 34, 868–877

Volz, S., Nöth, U., Jurcoane, A., Ziemann, U., Hattingen, E., and Deichmann, R. (2012). Quantitative proton density mapping: correcting the receiver sensitivity bias via pseudo proton densities. *Neuroimage* 63, 540–552
